# A detailed atomistic molecular simulation study on adsorption-based separation of CO_2_ using a porous coordination polymer

**DOI:** 10.1039/c8ra01408f

**Published:** 2018-04-17

**Authors:** Pezhman Zarabadi-Poor, Tomás Rocha-Rinza

**Affiliations:** Institute of Chemistry, National Autonomous University of Mexico, Circuito Exterior, Ciudad Universitaria Delegación Coyoacán C.P. 04510 Mexico City Mexico pzarabadip@gmail.com tomasrocharinza@gmail.com; CEITEC - Central European Institue of Technology, Masaryk University Kamenice 5 CZ-62500 Brno Czechia pezhman.zarabadi@ceitec.muni.cz

## Abstract

Emission of CO_2_ is considered as one of the sources of global warming. Besides its currently inevitable production *via* several processes such as fuel consumption, it also exists in some other gaseous mixtures like biogas. Separation of carbon dioxide using solid adsorbents, for example porous coordination polymers and metal–organic frameworks, is an interesting active area of separation science. In particular, we performed detailed molecular simulations to investigate the response of a recently reported cobalt-based, pillared-layer, porous polymer on the CO_2_ separation from biogas, natural gas, and flue gas. The effect of the coordinated water molecules to the open metal sites on the corresponding properties was studied and revealed enhanced results even in comparison with HKUST-1. Additionally, our results provide insights on the role of –NO_2_ groups on the applications examined herein. Overall this study offers valuable insights about secondary building units of the examined materials which we expect to prove useful in the enhancement of carbon dioxide separation and capture.

## Abstract 2

We examined a new pillared-layered Co-nitroimidazolate dicarboxylate porous coordination polymer for potential use in adsorption-based CO_2_ capture

## Graphical abstract

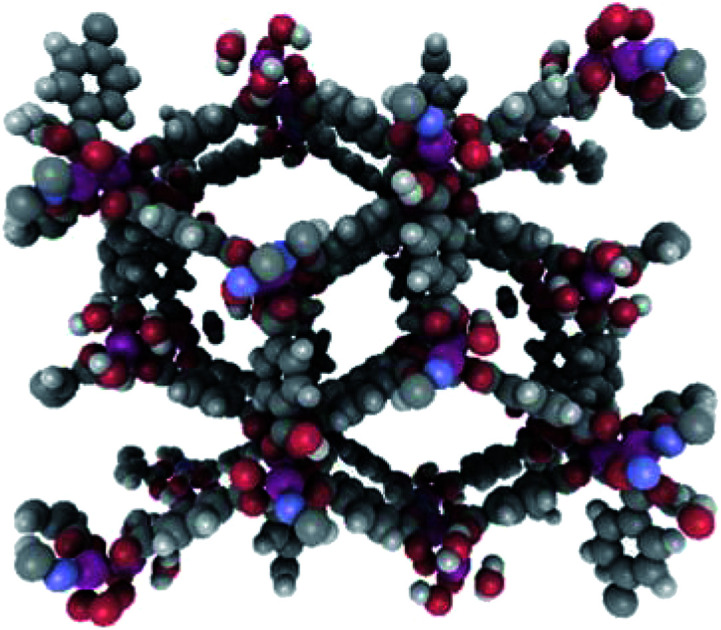

## Introduction

Carbon dioxide is known as one of the greenhouse gases which is emitted to the atmosphere in many different circumstances such as fuel consumption, and after that, it contributes to global warming. It can be present in several gas mixtures including biogas, natural gas, and post-combustion flue gas. Consequently, carbon capture and separation have raised significant interest in academia and industry. More specifically, it is of paramount importance to find plausible approaches to reduce the risks of carbon dioxide emission.^1^

When it comes to separation of CO_2_ from mixtures with other gases such as CH_4_ and N_2_, there are three main kind of materials used to overcome this task: solvent absorbers, membranes, and solid adsorbents.^2^ There are, of course, pros and cons for each method. Although amine solvents such as monoethanolamine have been used for more than 60 years, these methods suffer from significant energy demands for the regeneration step.^3,4^ On the other hand, despite the high selectivities and low energy requirements associated to the use of membranes, these processes are not the best choice for mixtures with low CO_2_ partial pressure.^5,6^ However, the last-mentioned approach started to be used widely because of the development of novel porous adsorbents. The regeneration of these adsorbents can be achieved by reducing the pressure or elevating the temperature, *i.e.*, Pressure-Swing Adsorption (PSA) and Temperature-Swing Adsorption (TSA), respectively. The particular case of PSA, in which its desorption pressure goes below ≈1 bar, is called Vacuum-Swing Adsorption (VSA) and it is useful when pressurising the feed stream is not applicable.^7^ Since the past decade, the usage of solid adsorbents has attracted a considerable amount of attention in several applications. Metal–organic Frameworks (MOFs), a family of Porous Coordination Polymers (PCP), have served many purposes such as gas storage^8^ and separation^9–12^ together with catalysis^13,14^ and different uses in biomedicine^15^ due to their fascinating structural properties like high surface area, porosity, thermal and chemical stability, and low density. MOFs became more interesting than zeolites and carbon-based solid adsorbents because these frameworks provide great flexibility through modular synthesis approach which creates the opportunity of producing materials with desired physical and chemical properties.^16^

There are several parameters that have proved relevant for the ability of PCPs and MOFs in the adsorption of carbon dioxide. One of these variables is the presence of Open Metal Sites (OMS). When one synthesizes MOFs through the combination of metal centres as Secondary Building Units (SBU) and organic linkers,^16^ solvent molecules, *e.g.* H_2_O, coordinate to the metal atoms of SBU and occupy the coordination sphere. However, the effect of this binding of solvent molecules (especially water) on the adsorption and separation ability of these materials has not been clearly established. On the one hand, there are investigations that suggest removal of coordination water molecules results in an increase of carbon dioxide capture due to the availability of a larger number of OMS to interact with this gas, while on the other hand, there are reports such as the study of Yazaydin that indicates that the presence of coordination water molecules can improve CO_2_ adsorption.^17^

Thus, we took the endeavour to determine the role of water molecules in the CO_2_ absorption of MOFs to obtain useful information for the design and synthesis of new systems utilized for carbon dioxide separation. For this purpose, we considered a novel pillared-layered network of Co-nitroimidazolate-dicarboxylate.^18^ Because this material exhibits channels and large cages, the authors also studied its gas uptake behaviour which presented promising results. This structure attracted our attention for further studies because it has water molecules coordinated to the SBU. We were also interested in testing the idea suggested by Guo *et al.*^18^ about the enhancement of CO_2_ uptake in virtue of the inclusion of nitro groups in these structures. Specifically, we studied:

• The detailed single component adsorption behaviour of the hydrated and dehydrated forms of the Co-nitroimidazolate-dicarboxylate based MOF discussed above denoted as 1 and 1′, respectively.

• The adsorption and separation of CO_2_ from gas mixtures representing biogas, natural gas, and flue gas. We choose these mixtures because they represent important systems from an industrial and academic perspective.

We employed atomistic molecular simulations methods plus adsorption theories to obtain unary and binary adsorption isotherms and related parameters to check the performance of the adsorbents addressed herein. We obtained insightful results on the effect of nitro moieties and coordination water molecules which are expected to be valuable in the design and synthesis of novel PCPs/MOFs in the industrial upgrading of different CO_2_-containing gaseous mixtures.

## Simulation details

Gas adsorption isotherms were simulated *via* the Grand Canonical Monte Carlo (GCMC)^19,20^ ensemble as implemented in the package Raspa.^21^ The atomic positions of the structures considered in this work were taken from X-ray single crystal CIF^18^ files and kept constant during the simulations. The coordination water molecules were carefully located and removed from the original CIF using CrystalMaker™ (ref. 22) to have a model for the completely evacuated structure. The non-bonded interactions between gaseous species and MOF atoms were described using a Lennard-Jones (L-J) 12-6 potential with no tail correction and a cut-off value of 12.0 Å. We also considered the Coulomb potential term for electrostatic interactions, into the expression1
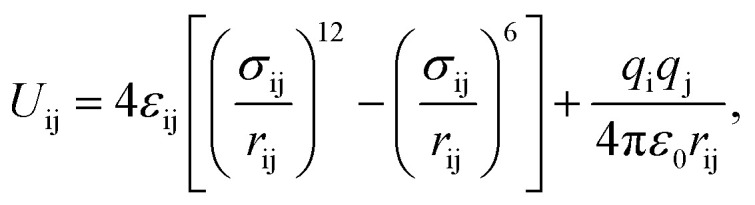
in which i and j are interacting atoms, *r*_ij_ denotes the distance between these species, *σ*_ij_ and *ε*_ij_ indicate the corresponding L-J potential parameters, and *q*_k_ is the partial charge of atom k. We benefited from the Ewald summation technique with a precision of 1 × 10^−6^ in modeling long-range electrostatic interactions. The Lorentz–Berthelot mixing rules^19^ were used to calculate the cross-term L-J parameters between atoms i and j as2
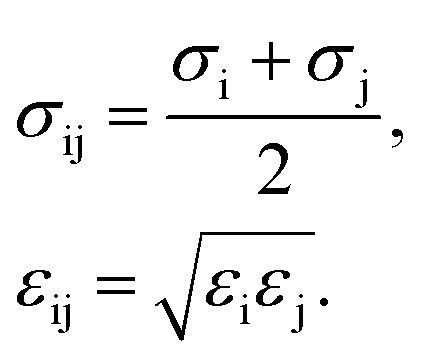


The L-J parameters for framework atoms appart from Co atoms (UFF^23^) were taken from the Dreiding generic force field^24^ and are presented in Table 1. The atomic partial charges of MOF structures were calculated by using the extended charge equilibration method developed by Wilmer *et al.*^25^ We used 10 × 10 × 10 unit cells to ensure that we included enough atoms in the charge calculation process to obtain accurate results and equal values of *q* on symmetry identical centres. The adsorbate molecules were modelled using the Siepmann's Transferable Potentials for Phase Equilibria Family of Force Fields (TraPPE). We considered the methane molecules as spherical non-charged particles^26^ which has already been compared with a 5-site model and proven to produce accurate results.^27^ We described the CO_2_ and N_2_ units as three-site rigid species^28^ (Table 1).

**Table 1:** UFF, DREIDING, and TraPPE forcefield parameters used for the molecular simulations performed in this investigation

Atom type	*ε* (K)	*σ* (Å)
Co	7.04	2.56
C	47.86	3.47
O	48.16	3.03
N	38.95	3.26
H	7.65	2.85
CO_2_(C)	27.00	2.80
CO_2_(O)	79.00	3.05
N_2_(N)	36.00	3.31
N_2_(COM)	0.00	0.00
CH_4_	148.00	3.73

The N_2_ molecule was represented by an L-J core at the center of mass (COM) with a point charge equal to −2*q* in which *q* = −0.482. The simulation box dimensions, *i.e. N* × *N* × *N*, were chosen considering that they should be higher than twice the above mentioned cut-off value to satisfy the minimum image convention. The gas adsorptions of single component and gas mixtures were simulated by considering 25 pressure points ranging from 0.001–100 bar to construct the adsorption isotherms and to have enough accurate results for curve fitting purposes. The pressure values were converted to fugacities which were used throughout the simulations to impose the equilibration between the system and the external gas container using the Peng–Robinson equation of state. The simulations at each pressure point included 5 × 10^5^ Monte Carlo (MC) cycles. The first half was used for equilibration and the rest for the computation of the average of thermodynamical properties. An MC cycle consists of *M* steps, *M* being the greater of 20 and the number of molecules at the beginning of each given simulation points. Insertion, deletion, translation, and rotation moves were used in all GCMC calculations. In addition, we utilized identity changes in the simulations of gaseous binary mixtures. The molar composition of binary mixtures, *i.e.*, biogas, natural and flue gas, are CO_2_ : CH_4_ (0.5 : 0.5), CO_2_ : CH_4_ (0.1 : 0.9), and CO_2_ : N_2_ (0.1 : 0.9), respectively. The isosteric heat of adsorption, *Q*_st_ was calculated based on the fluctuation method,^29^ that is, with the formula3
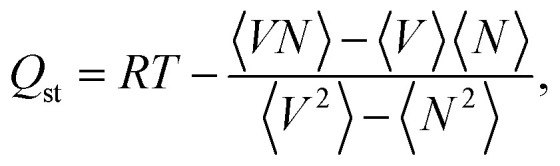
in which brackets represent ensemble averages, *V* stands for the potential energy, and *N* is the number of molecules.

## Results and discussion

### Structures

**Fig. 1:**
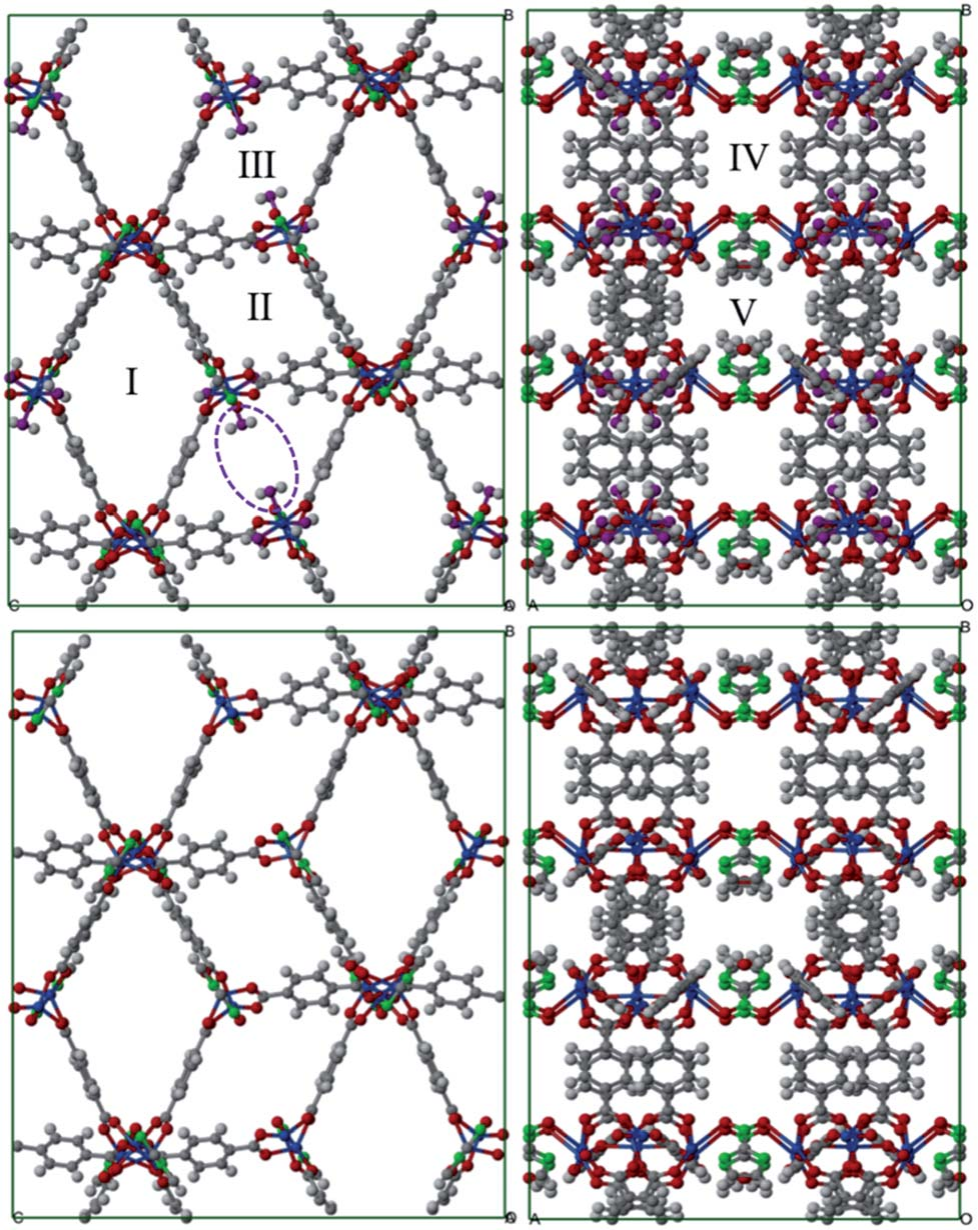
Structure representation of 1 (up) and 1′ (down). The used color code is as follows; dark grey: carbon, light grey: hydrogen, blue: cobalt, green: nitrogen, red: framework oxygen. The violet-dashed ellipse highlights the coordinated water oxygen. The different channel types are shown with Roman numerals.

Fig. 1 shows a representation of 1 and 1′. Guo *et al.*^18^ indicated the occurrence of different channel types within the structures as follows:

• Type I. They are the largest channels with no water molecules directly oriented toward the cavity of the framework.

• Types II and III. These two kinds of channels are quite similar except that coordination water molecules are oriented towards the channels of sort III.

• Type IV. This class of channel is aligned towards the *z* direction and includes the nitro moieties within pores.

• Type V. These channels are similar to Type IV but without nitro groups.

This identification of channels within the structures makes clear that the removal of water will mainly influence Type III. We also used the poreblazer algorithm^30^ to calculate the physical properties of both 1 and 1′. The corresponding results are reported in Table 2.

**Table 2:** Physical properties of 1 and 1′ (*V*_p_: pore volume, SA: surface area, PLD: pore limiting diameter, LCD: largest cavity diameter)

Structure	Density (g cm^−3^)	*V*_p_ (cm^3^ g^−1^)	SA (m^2^ g^−1^)	PLD (Å)	LCD (Å)
1	0.870	0.741	1587	5.74	8.38
1′	0.806	0.855	2026	6.41	10.16

We can observe that upon complete removal of coordination water molecules, all the properties shown in Table 2 (apart from the density) increase and consequently the evacuation of H_2_O can indeed influence the adsorption properties of the materials under study.

### Validation of force field

**Fig. 2:**
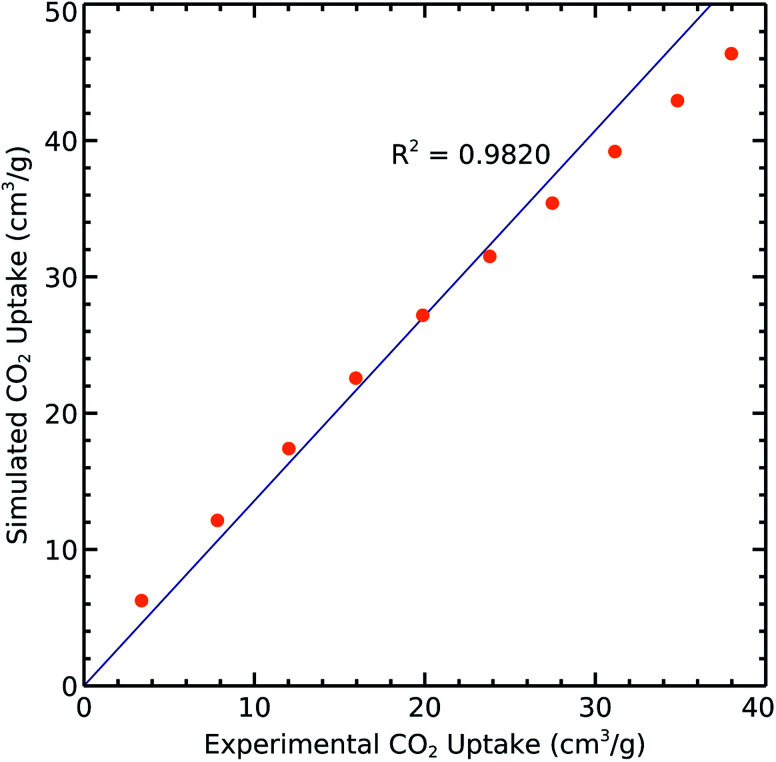
Experimental and simulated CO_2_ uptakes on 1 at 298 K.

We extracted the relevant reported experimental CO_2_ uptakes at 298 K from ref. 18 and compared them with our simulation results in order to validate the aforementioned methods and parameters. The resulting correlation with *R*^2^ = 0.9820 shows very good agreement between measured and calculated results (Fig. 2). However, the slight difference may come from the defects and impurities in experimental samples while we use perfect crystals for performing the simulations.

### Single component adsorption isotherms

**Fig. 3:**
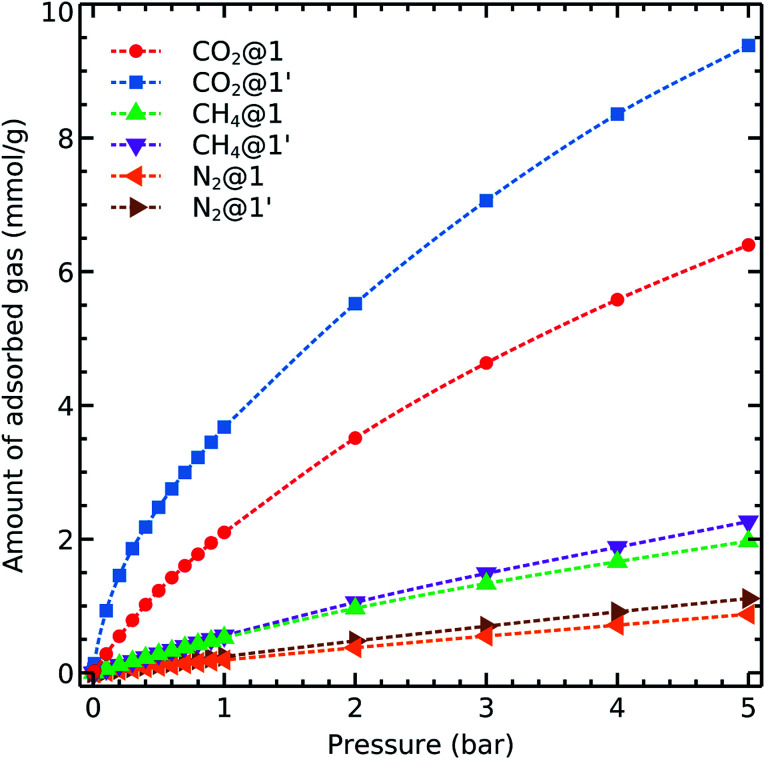
Simulated adsorption isotherms of CO_2_, CH_4_, and N_2_ on 1 and 1′ at 298 K.

We first consider the single component adsorption isotherms of CO_2_, CH_4_ and N_2_ on 1 and 1′ shown in Fig. 3. We see that the complete removal of coordination water molecules from the metal centre resulted in a significant improvement of the carbon dioxide uptake. This effect evidences the role of OMS in CO_2_ adsorption.

Moreover, we also note two remarkable trends concerning the adsorption isotherms of CH_4_ and N_2_:

• The gas uptake of both gases is notably lower than that of CO_2_, and

• The adsorption behaviour and capacity of methane and nitrogen do not change upon activation.

Both observations suggest that 1 and 1′ are promising materials for adsorption-based separation of CO_2_ from different gas mixtures containing nitrogen and methane as its uptake is significantly higher than it is for the two last-mentioned gases. Furthermore,the CO_2_ uptake can be enhanced through removal of coordinated water molecules to the metal centres. We emphasise that both CO_2_ and N_2_ interact with the MOFs *via* Coulomb and van der Waals contacts and that the availability of OMS increases CO_2_ adsorption but do not rise N_2_ uptake. These differences can be related to the large quadrupole moment of carbon dioxide as compared with that of nitrogen.

**Fig. 4:**
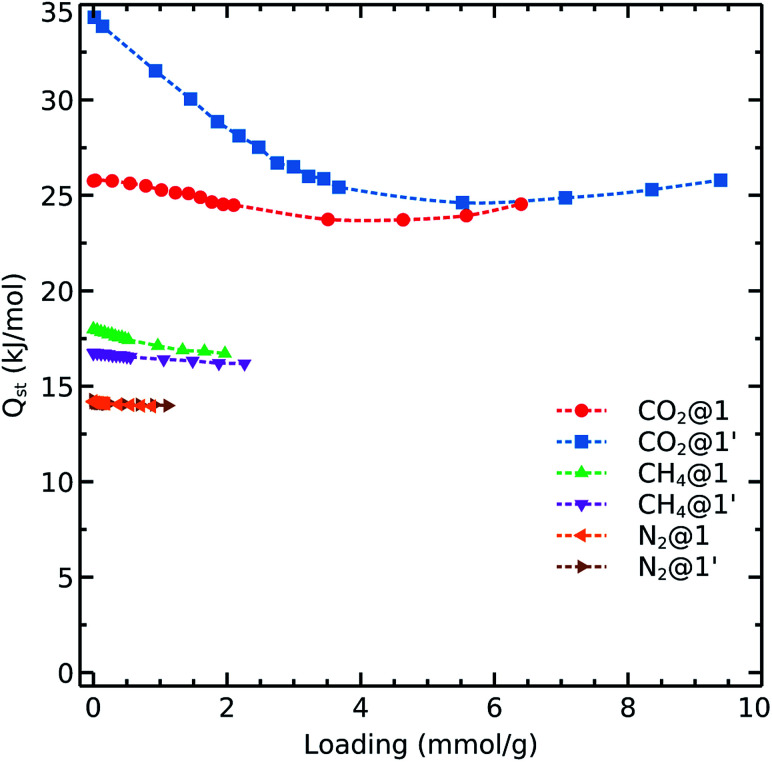
Calculated CO_2_, CH_4_, and N_2_ isosteric heats of adsorption on 1 and 1′ at 298 K.

**Fig. 5:**
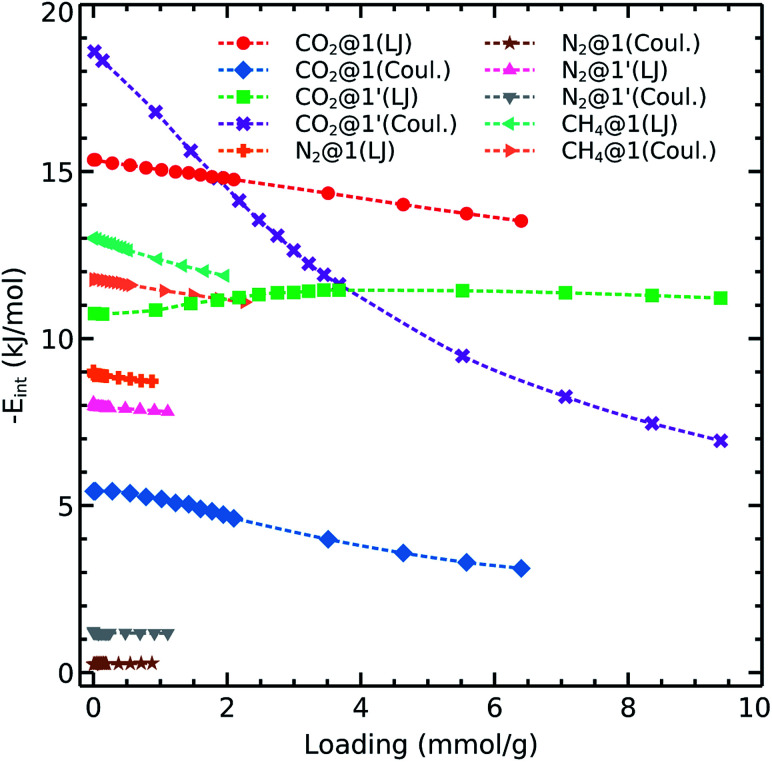
Interaction energies of CO_2_, N_2_, and CH_4_ with 1 and 1′ as computed with eqn (1).

To get further insights into the interaction nature of these gases with the studied frameworks, we monitored the isosteric heat of adsorption and host–adsorbate interaction energies as shown in Fig. 4 and 5. The decreasing order of *Q*_st_ for the different gases is:

4 *Q*_st_(CO_2_) > *Q*_st_(CH_4_) > *Q*_st_(N_2_)

Carbon dioxide exhibits the strongest affinity for the available adsorption sites with its isosteric heat of adsorption being almost 10–15 kJ mol^−1^ higher than those for CH_4_ and N_2_. We see that *Q*_st_(CO_2_) in 1′ exhibits a different behavior as compared to that observed in 1. The carbon dioxide heat of adsorption decays in the dehydrated system up to loadings corresponding to 1 bar pressure (*i.e.*, 48 CO_2_ molecule per unit cell which contains 56 cobalt atoms, in an almost 1 : 1 ratio). Therefore, it again can be concluded that the OMS play a relevant role in the adsorption of CO_2_ up to 1 bar. We note that the OMSs are occupied above this pressure as the heat of adsorption becomes steady for both 1 and 1′.

As shown in eqn (1), we are considering van der Waals and Coulomb terms for the interaction energy between two fragments. The examination of each term provides useful information on the nature of contact. The methane molecule has neither a permanent dipole nor quadrupole moment and thus we consider only the L-J contribution. We observe that the CH_4_–host interaction energy is about 12–13 kJ mol^−1^ and it does not change by increasing the loading. This circumstance shows that methane adsorption sites are still available even at loadings which correspond to pressures as high as 5 bar. We also notice the same trend for nitrogen adsorption in both 1 and 1′. The relevant van der Waals interaction energy is around 8–9 kJ mol^−1^ while the coulombic term lies below 1 kJ mol^−1^ in the absence of OMS and increases up to unity upon activation.

Concerning the adsorption of CO_2_ in 1, this species exhibits L-J and coulombic interaction energies of 15 kJ mol^−1^ and 4–6 kJ mol^−1^, respectively. Interestingly, when we remove the coordination water molecules, the L-J contribution decreases to 11–12 kJ mol^−1^ while the coulombic component increases up to ≈20 kJ mol^−1^. This effect is consistent with the observations discussed above on the carbon dioxide isosteric heat of adsorption which decreases up to 8 kJ mol^−1^ with the increase of loading.

### Binary mixture adsorption

**Fig. 6:**
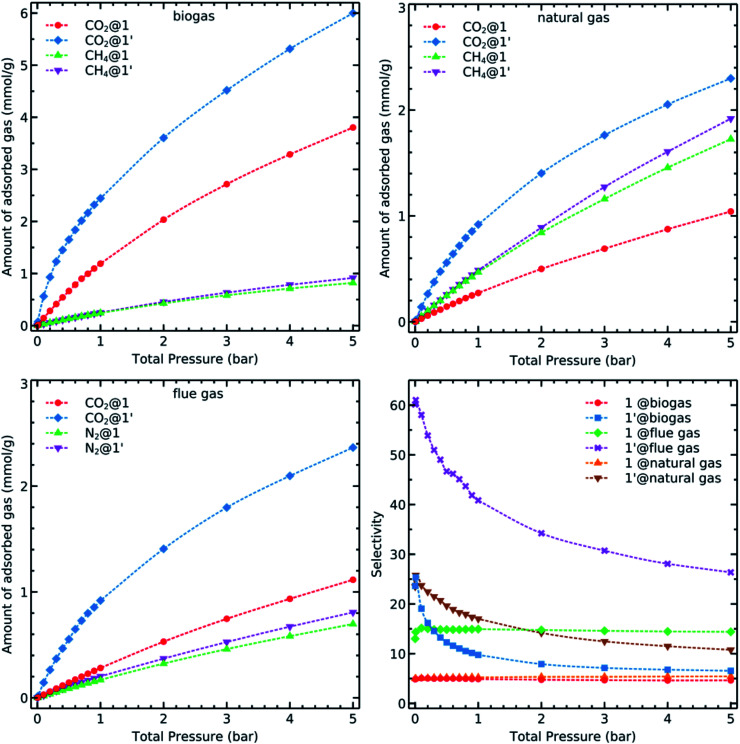
Simulated binary adsorption isotherms of CO_2_, CH_4_ and N_2_ in biogas (CO_2_ : CH_4_, 0.5 : 0.5), natural gas (CO_2_ : CH_4_, 0.1 : 0.9), and flue gas (CO_2_ : N_2_, 0.1 : 0.9) at 298 K.

We have discussed so far the adsorption of unary gases on 1 and 1′. Now, we examine the adsorption behaviour of the gases addressed herein when they are mixed with each other. Although we could consider many combinations based on the molar fraction ratios and number of components, we investigated three mixtures which are representative of industrially relevant systems: biogas, natural and flue gases^2^ with the molar compositions mentioned at the end of the “Simulation Details” section. We simulated the adsorption isotherms of binary mixtures using the GCMC (Fig. 6).

CO_2_ uptake of 1′ in all cases is higher than it is in 1 due to the availability of OMS in the former system. Carbon dioxide is less adsorbed in natural and flue gases because of the smaller amount of this component in these mixtures. Every studied material has, however, a similar behaviour concerning the adsorption of methane and nitrogen. Although the adsorption isotherms of mixtures are important in understanding the capacity of a given material for carbon dioxide capture and detachment, it is necessary to further analyse the calculated data to obtain more detailed insights on the capability of these materials for adsorption-based separation of CO_2_ from a given mixture. There are several parameters that we should consider for this purpose. For example, the CO_2_ selectivity of the adsorbent towards methane or nitrogen as obtained through the equation5
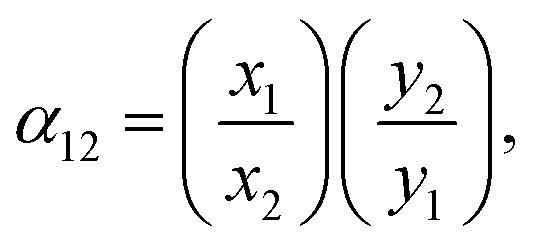
where 1 stands for CO_2_ and 2 denotes CH_4_ or N_2_, *x* refers to the adsorbed amount in mmol g^−1^ and *y* is the mole fraction in gas phase.

We applied the selectivity formula on simulated mixture adsorption isotherms and report the outcome graphs in Fig. 6. The presence of OMS and their previously mentioned influence on the adsorption of CO_2_, especially in lower loadings, with 1 present steady values within the whole examined pressure range. The selectivity *α*_CO_2_/CH_4__ for biogas and natural gas has almost a constant value at low loading while it decays faster in biogas due to the presence of more CO_2_ molecules. This condition results in a faster saturation of the available OMS. On the other hand, 1′ has a twice higher initial selectivity for CO_2_ over N_2_ despite a larger amount of N_2_ molecules in the flue gas mixture. These observations might be related to the increasingly ordered kinetic diameter values of: CO_2_(3.30 Å) < N_2_ (3.64 Å) < CH_4_ (3.80 Å), a factor which can be advantageous for the carbon dioxide molecules to reach adsorption sites more easily than methane and nitrogen.

Besides selectivity, there are other parameters that have an important effect on the separation performance of adsorbents. Snurr and his co-workers summarized and introduced different of these descriptors^2,31^ such as the working capacity (Δ*N*), the regenerability factor (*R*) and the adsorption performance score (APS) which are defined as

6 Δ*N*_1_ = *N*^ads^_1_ − *N*^des^_1_,

7
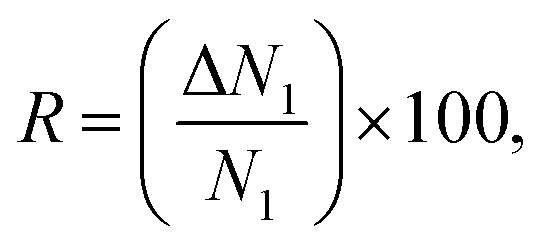

8 APS = (*α*_12_) × (Δ*N*_1_),

respectively. In eqn (6)–(8)*N*, 1, and 2 indicate uptake at adsorption (ads) or desorption (des) pressure, CO_2_, and (CH_4_ or N_2_) in the same order.

The occurrence of OMS leads to a significant improvement in all the adsorption parameters apart from *R*. The values of this indicator decrease because of the strong interaction of CO_2_ with the 1′ framework which impairs the removal of the adsorbed gases. However, this parameter is still in a reasonable range. We compare now our results with data of HKUST-1 (ref. 2) which is known as a reference MOF with a good performance in CO_2_ separation.

Table 3 shows that in all cases 1 presents much lower values in comparison with HKUST-1 but once we remove all solvent molecules, the parameters enhance siginificantly. The MOF 1′ shows promising results for the separation of CO_2_ from biogas and natural gas through PSA processes. Likewise to HKUST-1, the availability of OMS results in a better interaction with carbon dioxide molecules. The best improvements occur in VSA conditions for biogas, *i.e.*, case 3 in Table 3, which provides better performance even in comparison with HKUST-1. The values corresponding to this last statement are bolded in the same chart. The values of *R* indicate that slightly lower uptakes at VSA adsorption pressures, *e.g.*, 1 bar, brings the possibility of an easier adsorption sites regeneration. However, the selectivity of 1′ toward CO_2_ is most likely attributed to the higher uptake of carbon dioxide due to the presence of OMS, as indicated by the methane uptake at 1 bar which in both cases 2 and 3 is around 0.25 mmol g^−1^, resulting in an almost doubled APS for 1′.

**Table 3:** Adsorbent evaluation parameters for 1, 1′, and HKUST-1 ^2^

Structure	*N*_1_ (mmol g^−1^)	Δ*N*_1_ (mmol g^−1^)	*R* (%)	*α*	APS
**Case 1: natural gas (PSA)**					
1	1.04	0.77	74.0	5.4	4.1
1′	2.30	1.38	60.0	10.8	14.9
HKUST-1	2.70	1.70	63.0	10.0	17.0

**Case 2: biogas (PSA)**					
1	3.80	2.61	68.7	4.6	12.0
1′	6.00	3.56	59.3	6.6	23.5
HKUST-1	8.01	5.34	66.7	4.9	26.2

**Case 3: biogas (VSA)**					
1	1.19	1.05	88.2	4.9	5.1
1′	2.44	1.88	**77.0**	**9.8**	**18.4**
HKUST-1	2.81	1.90	67.5	5.5	10.4

### Adsorbate density maps

**Fig. 7:**
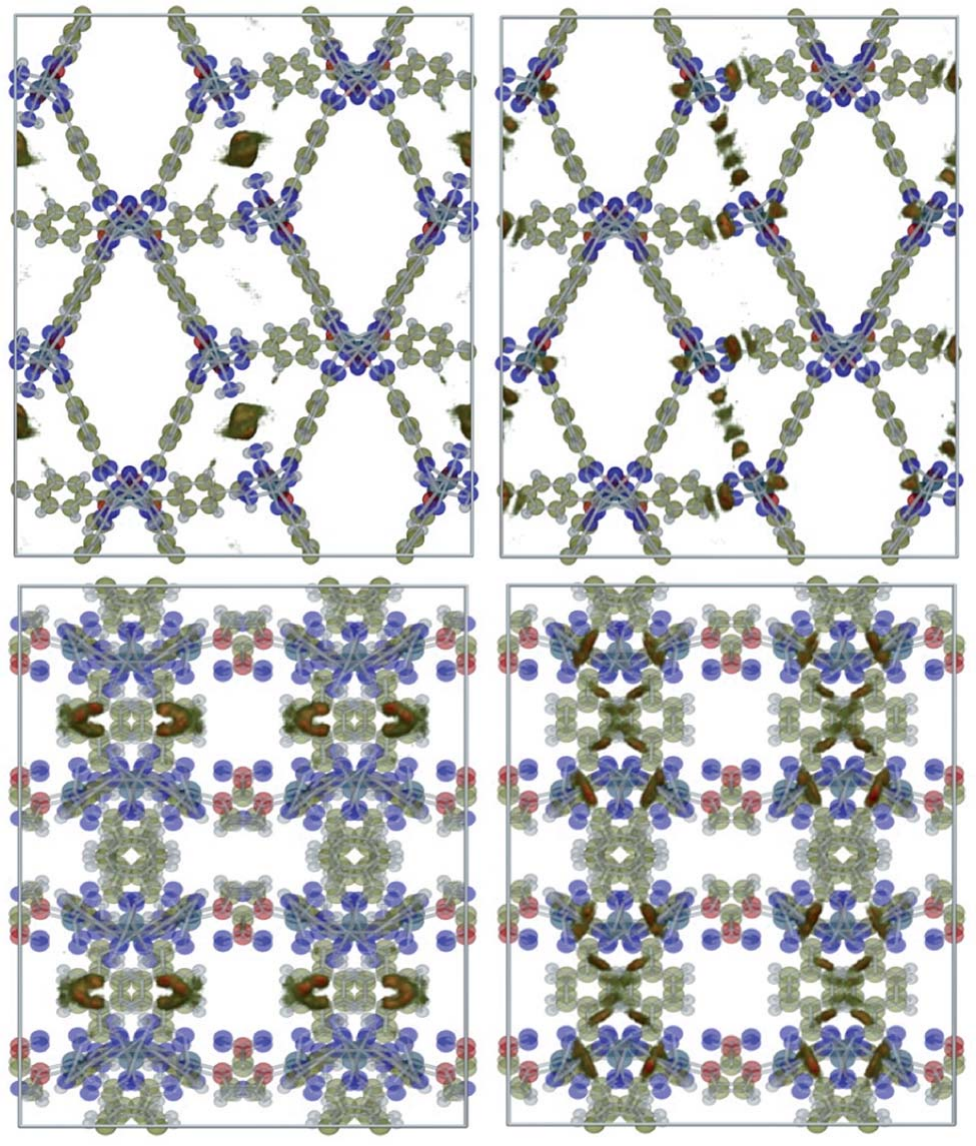
Density plots of CO_2_ in biogas within 1 (left) and 1′ (right) simulated at 298 K and 5 bar.

**Fig. 8:**
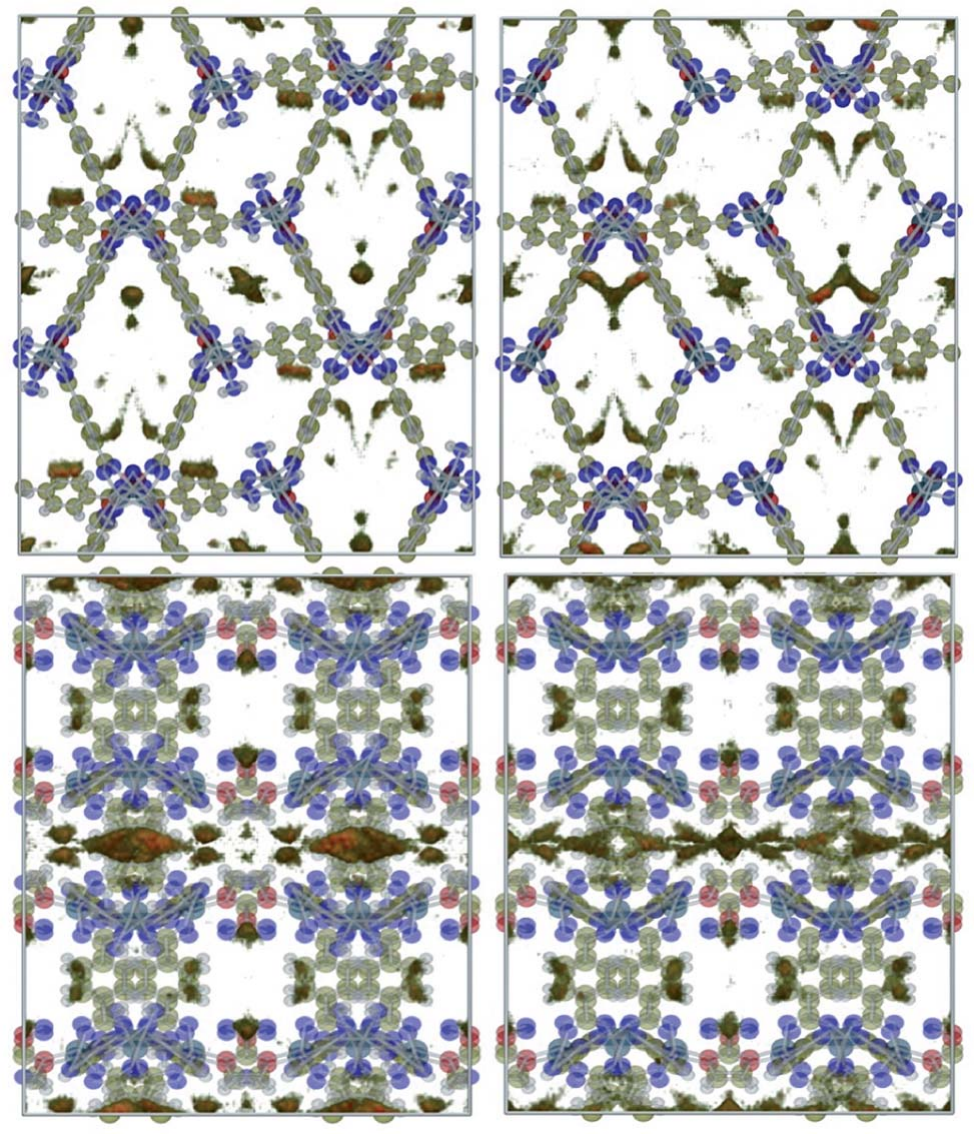
Density plots of CH_4_ in biogas within 1 (left) and 1′ (right) simulated at 298 K and 5 bar.

Guo *et al.*^18^ suggested that the presence of nitro groups in Type IV channels might enhance CO_2_ adsorption. To test this statement, we investigated the density plots of CO_2_ and CH_4_ in 1 and 1′ which are obtained using RASPA by dividing the unit cell into voxels and calculating the probability density of adsorbates in each voxel as shown in Fig. 7 and 8. The former plot shows that in both 1 and 1′ CO_2_ molecules are adsorbed in Type III channels. The only difference is that in the 1, carbon dioxide units interact with coordination water molecules whose removal provides the chance for more numerous and stronger interactions with cobalt open sites. The same holds true for natural and flue gases which have only a 0.1 molar ratio of CO_2_.

We also explored the possible adsorption sites for methane molecules and found out that they are adsorbed in all of the types of examined channels except Type III. Because (i) the interaction of methane with the framework occurs solely through van der Waals contacts, and (ii) the interacting atoms construct the skeleton of the framework, we propose to use SBU with higher affinity to CO_2_ as well as trying to provide OMS in the final product to enhance carbon dioxide capture and separation.

Finally, the comparison of the properties of 1 and 1′ reveal that the differences in their performance for CO_2_ adsorption and separation can be understood in simple physical interaction terms and OMS. Our results indicate that the NO_2_ groups are not the adsorption sites for the CO_2_ molecules and hence polar functional groups are not to be held responsible for the different behaviour of the MOFs studied herein.

## Conclusions and prospects

We performed detailed molecular simulations on a Co-nitroimidazolate-dicarboxylate pillared layered polymer and its water-removed structure to investigate the role of coordinated H_2_O molecules in adsorption-based separations of CO_2_. We concluded that the performance of the material is enhanced by removal of H_2_O molecules from the cobalt centres to make the secondary building units available to carry out the process. In addition, our results suggest the nitro groups has unimportant effects on the adsorption and separation of carbon dioxide in opposition with the suggestion made in the original report of 1 concerning this matter. Altogether, we suggest that the overall performance of these materials can be enhanced by using SBU with a high affinity for CO_2_ and that the design should include a large number of open metal sites for the adsorption to take place.

## Conflicts of interest

There are no conflicts to declare.

## Supplementary Material
